# Patterns of Weight Change Trajectories and Treatment Response in an Integrated Adult Primary Care Weight Management Practice

**DOI:** 10.1002/osp4.70045

**Published:** 2025-01-13

**Authors:** Anita Ganti, Shanna Tucker, Afua Takyi, Musarrat Nahid, Alexa Bickhart, Debra Katz‐Feigenbaum, Erica Phillips

**Affiliations:** ^1^ Division of General Internal Medicine Weill Cornell Medicine New York New York USA; ^2^ Department of Medicine NYU Grossman School of Medicine New York New York USA; ^3^ Comprehensive Weight Control Center Weill Cornell Medicine New York New York USA; ^4^ Outpatient Nutrition, Diabetes Education and Breastfeeding Support Services NewYork‐Presbyterian Hospital New York New York USA

## Abstract

**Introduction:**

Given the significant interindividual variable responses to interventions for obesity, the early identification of factors associated with a differential in weight loss would benefit real‐world approaches in clinical practice.

**Objective:**

This study evaluated the factors associated with individual variability in response to enrolling in a weight management program integrated into an academic‐based primary care practice.

**Methods:**

Data were retrospectively collected and analyzed for patients referred to a primary care‐based weight management practice between 2012 and 2020. A mixed‐model, semi‐parametric group‐based modeling approach was used to identify group membership and explore weight change trajectories over 18 months, as measured by the percent of initial body weight loss and the probability of losing at least 5% of initial body weight (IBW).

**Results:**

Three hundred ninety‐three patients were included in the study; the median age was 53 years, 84% female, 40% self‐identified as non‐Hispanic Black, and about one‐third white. Among those, 374 had sufficient follow‐up data for group‐based modeling. Four groups were identified and named: “*Weight Gainers”* added 1.3% of IBW; *“Initial Resistors”* lost 6.7% of IBW; “*Early Maintainers*” lost 7.1% of IBW; and “*Steady Achievers*” lost 15% of IBW. Weight change in all groups was over 18 months. The probability of losing 5% IBW was described by three groups: *“Minimal Late Responders,” “Moderate Responders,” and “High Early Responders.”* Younger age, non‐Hispanic Black race, fewer follow‐up visits, and lower proportion prescribed two or more anti‐obesity medications (AOMs) simultaneously were associated with a lower probability of achieving 5% IBW.

**Conclusions:**

Compared to the other groups, Weight Gainers and Minimal Late Responders had a distinct trajectory associated with two modifiable factors: the number of treatment visits and AOMs. Tailored interventions targeting these factors early may increase the probability of meaningful weight loss.

## Graphical abstract

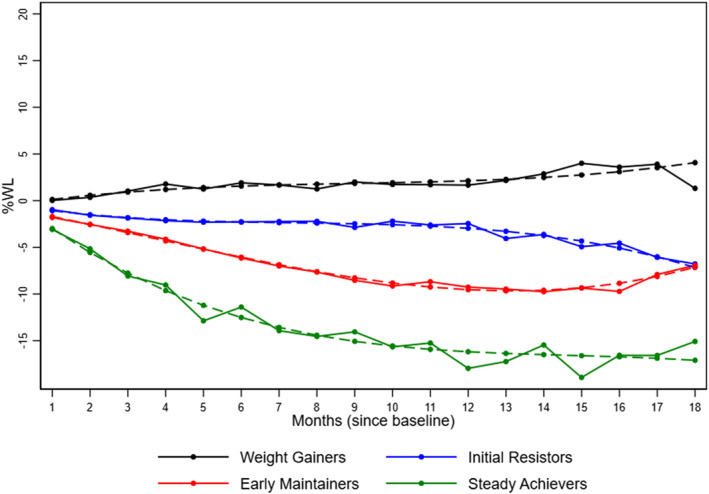

## 1 Introduction

Among adults aged 20 and over, the age‐adjusted prevalence of obesity is now almost 50% in the US population [1]. The ubiquity of this condition and the clinical significance of its weight‐related health conditions represent significant challenges for the primary care setting. Obesity treatment offered through primary care practices has the potential for a broad population reach. However, despite frequent contact with adults living with obesity and providers' recognition of the importance of weight loss, provider‐delivered weight loss counseling remains limited in primary care settings [2, 3, 4, 5, 6].

Most primary care providers acknowledge that they have limited training, time, and confidence in the effectiveness of behavioral counseling or pharmacological management of obesity to incorporate it into typical office visits [7, 8, 9]. In a 2019 review of primary care interventions for treating obesity, Tronieri et al. found no studies in which PCPs, working alone or with trained interventionists, provided intensive behavioral counseling as recommended by the Centers for Medicare and Medicaid Services (CMS) [10, 11]. Among the studies in the review, three trials provided approximately monthly brief counseling visits delivered by trained medical assistants collaborating with PCPs. Mean weight losses at 6 months ranged from 3.5 to 4.4 kg, with 48% of participants in one study losing ≥ 5% of baseline weight. Mean weight losses in at least two trials declined over follow‐up, and smaller 12–24‐month losses were observed when PCPs, working alone, provided quarterly or less frequent weight loss counseling [12]. The limited training and confidence in the management of obesity results in low utilization of weight management treatment strategies, as evidenced by a study conducted at a large academic medical center that found that over 2 years, weight management therapy utilization for patients with a diagnosis of obesity was only 7.1%. The probability of achieving 5% weight loss or more was increased in patients who were exposed to weight management therapy compared with those who were not exposed to any weight management therapy. Of note, only 3.4% of over 17,000 patients were prescribed an anti‐obesity medication [13].

Currently, one medication is FDA‐approved for short‐term weight loss, Phentermine (Adipex‐P, Lomaira), while six medications are approved for long‐term weight loss. The latter include Orlistat (Alli, Xenical), Phentermine‐topiramate (Qsymia), naltrexone‐bupropion‐ (Contrave), liraglutide (Saxenda), semaglutide (Wegovy), and most recently tirzepatide (Zepbound) [14]. Although these medications or components thereof are commonly prescribed for treating other chronic medical conditions, such as diabetes, migraine headaches, attention deficit disorders, or depression, many primary care physicians are hesitant to prescribe them for the sole purpose of treating obesity due to lack of knowledge of the safety profile or their long term effectiveness [15, 16]. Furthermore, many insurance companies do not provide coverage for the cost of these medications, especially if the patient has no weight‐related health conditions. A study by Gomez et al. that evaluated insurance coverage for anti‐obesity medicines in the United States revealed that “*among 136 marketplace health insurance plans, 11% had some coverage for certain drugs in nine states, Medicaid programs have drug coverage only in 7 states, and Medicare excluded weight loss drugs from coverage.*” [17] Thus, while the Center for Medicare and Medicaid's decision to reimburse for intensive obesity behavioral counseling has reduced some provider‐level barriers, there remain significant barriers regarding implementing all evidence‐based treatment options among those who qualify. Most recently, in March 2024, the FDA approved Wegovy (semaglutide) to reduce the risk of cardiovascular death, heart attack, and stroke in adults with cardiovascular disease and either overweight or obese [18, 19]. At the inception of our study, Medicare did not cover Wegovy to prevent cardiovascular death. While this expanded coverage criteria will allow additional patients to benefit from anti‐obesity medications, many patients remain restricted from accessing these beneficial and, at times, life‐changing medications.

Given the well‐established evidence that weight loss is heterogeneous, there remains a need to identify factors associated with less successful weight loss attempts. The early identification of individuals at most risk for limited to no weight loss over time could lead to tailored treatment plans to meet their needs. Hence, this study aims to describe the demographic and clinical characteristics associated with weight loss patterns over 18 months among adults referred to a weight management program integrated into an academic primary care practice.

## 2 Materials and Methods

### 2.1 Study Population

A retrospective analysis used data from a clinical practice registry of patients referred to a weight management program integrated into an academic primary care practice. Patients with a body mass index (BMI) ≥ 30 (or BMI ≥ 27 with an associated weight‐related condition) who had an index initial visit and at least one follow‐up visit to the practice for weight management between July 2012 and January 2020 were identified. This search resulted in 393 unique patients with a cumulative total of 2588 outpatient office visits where weight was recorded during the follow‐up period.

### 2.2 Description of the Weight Management Program

Referred patients are scheduled for monthly in‐person visits for at least 6 months with a board‐certified obesity medicine specialist and a registered dietitian. After the first 6 months, patients who achieve clinically meaningful weight loss, defined as a loss of at least 5% of their initial body weight, may shift to an appointment cadence of every 6–8 weeks for up to 12 months. However, patients also have the option to continue monthly visits based on their preferences. Generally, patients with less than 5% weight loss who wish to continue in the program are encouraged to maintain at least monthly visits. The program's primary goal is to achieve a clinically meaningful weight loss goal of at least 5% by 6 months and a 6%–10% loss by 12 months. Secondary programmatic goals include improvement in self‐monitored blood sugars or laboratory‐based hemoglobin A1c in patients with type II diabetes or pre‐diabetes (defined as hemoglobin A1c between 5.6% and 6.4%); decrease in systolic or diastolic blood pressure in patients with known hypertension; decrease in lipid levels (total cholesterol, triglycerides or LDL); decrease in self‐reported joint pain; and improved self‐reported sleep patterns (less nighttime awakening).

The registered dietitian calculates a patient's resting metabolic rate at the first practice visit using the Mifflin‐St Jeor equation [20]. A daily caloric goal of 250–500 caloric deficits is generally recommended depending on a patient's baseline resting metabolic rate. The macronutrient content of the meals is guided by MyPlatePlanner [21] and personalized based on age, sex, food preferences, allergies, and metabolic health conditions, such as diabetes or chronic kidney disease. Patients are encouraged to monitor their dietary intake at least 3 days a week (2 weekdays and one weekend) by recording everything they eat, drink, and portion sizes using dietary diary sheets given to them at the initial visit. Patients can also track food and beverage consumption using a free calorie and exercise counter application that automatically sends the patient's data to the practice team for weekly review. Patients are encouraged to eat three meals and 0 to 2 portion‐controlled snacks a day, if needed. Patients are also advised to achieve at least 150 min of moderate physical activity weekly. For patients with musculoskeletal disease (e.g., osteoarthritis) that impacts their ability to be more physically active, a request is made to the primary care provider to refer the patient to physical therapy to obtain a safe, individualized aerobic and strengthening program that can be done at home.

Anti‐obesity medications (AOMs) are prescribed by the obesity medicine specialist based on evidence‐based guidelines for individuals with a body mass index (BMI) ≥ 30 kg/m^2^ or those with a BMI ≥ 27 kg/m^2^ and diagnosed with at least one weight‐related condition such as hypertension or type 2 diabetes mellitus. AOMs are generally not initiated at the first visit, but rather, patients are informed about any chronic medications they may be on that can potentiate weight gain or make it difficult to lose weight (e.g., certain beta blockers) [22, 23]. Information regarding the conflicting medications and, where possible, information regarding alternative medicines is sent to the prescribing physician as part of the initial practice consultative visit note. This is a critical treatment step as population‐level data demonstrate that more than 20% of US adults are on weight‐inducing medications [24]. A discussion about the role of AOMs in the treatment plan is conducted during the second program visit. In preparation for that visit, patients are asked to review a handout developed by the program, which explains on and off‐label usage of medications, how the medication works, what kind of weight loss can be expected, typical side effects, and estimated monthly costs when not covered by insurance. Patients are also advised to contact their health insurance plan to understand if they have prescription benefit coverage for FDA‐approved AOMs and the insurance's preferred formulary. Within the practice, prescribing medications is tailored to each patient's unique needs, other concomitant chronic health conditions, and out‐of‐pocket costs.

At each follow‐up visit, body weight is measured, a review of physiological responses (e.g., decreased hunger) to AOMs is conducted, and dietary and physical activity counseling is conducted. If at least a three to 5% mean weight loss is not achieved after 3 months of prescribing an AOM (e.g., phentermine‐topiramate, naltrexone‐bupropion) at the standard treatment dose, the medication is discontinued, and alternative medicines are prescribed.

### 2.3 Study Variables

The primary outcome of weight was tracked approximately every 4 weeks for at least 18 months after the initial visit. Patient demographics (age, gender, race, ethnicity, employment, and health insurance), anthropometrics (height [in], weight [lbs], BMI [weight in kg/height in m^2^]), laboratory tests (hemoglobin A1c, low‐density lipoprotein, high‐density lipoprotein, total cholesterol, and triglycerides), and weight‐related health conditions defined according to ICD 10 codes were abstracted from the electronic health record (EHR). We also measured patient care variables and health outcomes, including the number of follow‐up visits, number of visits with self‐reported changes in diet grouped into six categories (e.g., reduced consumption of sweetened beverages), number of visits with self‐reported changes in physical activity grouped into four categories (e.g., walked more), medications initiated for weight loss, changes in medication at follow‐up visits (e.g. increased, decreased, discontinued, new medication added) and improvements in secondary outcomes (e.g., improved blood pressure control) at follow up visits.

### 2.4 Statistical Analysis

A mixed‐model, semi‐parametric group‐based modeling approach was used to identify latent groups and explore trajectories of weight change over 18 months by percent of initial body weight loss and the probability of losing at least 5% of initial body weight. Group‐based trajectory modeling (GBTM) assumes that the population comprises distinct groups, each with a different underlying trajectory and without any variation between individuals in the same group [24].

SAS macro ‘PROC TRAJ’ was used to conduct the analysis; the approach used by ‘Proc Traj’ complements two well‐known methods for analyzing developmental trajectories: hierarchical modeling and latent growth curve modeling. Fit statistics such as BIC, log of Bayes Factor, subject matter knowledge, and visual inspection of trajectories were used to identify the optimal number of trajectory groups. After selecting the model with the most suitable number of groups, differences in demographical (age, gender, race) and clinical characteristics (BMI at baseline, prior bariatric surgery, metabolic syndrome, number of weight management follow‐up visits, and proportion prescribed two or more weight loss medications) among groups were evaluated using Kruskal‐Wallis tests or Fisher's Exact tests for continuous and categorical variables, respectively. As is customary with group‐based analysis, the investigators assigned names to the newly identified groups based on the visual patterns and the nature of the outcome variable. Data were analyzed using SAS 9.4 (SAS institute, Cary, NC, USA) and Stata 17 MP (StataCorp LLC, College Station, TX, USA) software.

### 2.5 Ethical Considerations

The Institutional Review Board of Weill Cornell Medicine approved the study.

## 3 Results

### 3.1 Characteristics of the Study Population

Patient characteristics (*N* = 393) are presented in Table 1. The median age was 50 years [IQR 42, 62], with 84% of the cohort being women. More than half of the patients self‐identified as non‐Hispanic Black (41%) or Hispanic (31%), about half were employed (49.3%), and 62% had Medicaid and Medicare as their primary health insurance. The median BMI was 39.2 kg/m^2^, with 46% of patients having class III obesity (BMI ≥ 40 kg/m^2^). Hypertension (48%) was the most common weight‐related health condition, followed by dyslipidemia (37%), pre‐diabetes (32%), depression (30%), and diabetes mellitus (20%).

**TABLE 1:** Baseline characteristics of patients in the weight management program (*n* = 393).

Demographics	
Median age in years (IQR)	53 (42, 62)
Female sex, *n* (%)	329 (83.7)
Self‐reported race, *n* (%)	
Black	161 (41.0)
White	113 (28.8)
More than one race	83 (21.1)
Other/unspecified	28 (7.1)
Asian	6 (1.5)
American Indian/ Alaska Native	2 (0.5)
Self‐reported ethnicity, *n* (%)	
Hispanic	122 (31.0)
Non‐Hispanic/unknown	271 (69.0)
Health insurance, *n* (%)	
Medicaid	190 (48.5)
Commercial	118 (30.1)
Medicare	55 (14.0)
Medicaid/medicare	22 (5.6)
Other state‐funded	7 (1.8)
Employed, *n* (%)	176 (49.3)

Clinical characteristics	
Weight in lbs, median (IQR)	232 (206, 274)
Height inches, median (IQR)	64.96 (63, 67)
Body mass index kg/m^2^, median (IQR)	39.2 (35.3, 44.9)
Hemoglobin A1c, median (IQR)	5.8 (5.5, 6.2)
Lipids mg/dl, median (IQR)	
Low‐density lipoprotein (LDL)	108 (88,130)
High‐density lipoprotein (HDL)	50 (41, 61)
Total cholesterol	185 (158, 212)
Triglycerides	112 (79, 165)
Medical conditions	
Hypertension	187 (47.6)
Dyslipidemia	144 (36.6)
Pre‐diabetes (HbA1c 5.7%–6.4%)	126 (32.1)
Depression	119 (30.3)
Obstructive sleep apnea	74 (18.8)
Diabetes mellitus	73 (18.6)
Metabolic syndrome	42 (10.7)
History of bariatric surgery	30 (7.9)

*Note:* Missing data: insurance, *n* = 392; employed, *n* = 357; hemoglobin A1c, *n* = 352; ldl, *n* = 357; hdl, *n* = 359; triglycerides, *n* = 359, history bariatric surgery, *n* = 380.

As shown in Table 2, the most common dietary change made by patients (43%) was selecting foods lower in glycemic index (i.e., complex rather than refined carbohydrates and processed foods). This was followed by portion control (27%), reducing the consumption of sweetened beverages (20%), and reducing the number of snacks consumed daily (20%). The most common form of physical activity was walking (43%) or going to the gym (23%). The top four medications prescribed for weight loss were metformin (56%), topiramate (24%), bupropion (19%), and phentermine (16%). A little more than a third (38%) of patients were prescribed either a combination pill (e.g., naltrexone‐bupropion or phentermine‐topiramate) or two individual medications to be used together as a combination pill (e.g., topiramate and phentermine). At follow‐up visits, about one in four patients (27%) reported improvements in how their clothes fit, 15% reported improvements in their overall energy level, 5% had fewer nighttime awakenings, and 4% reported a reduction in general joint pain.

**TABLE 2:** Changes in health behaviors, obesity pharmacotherapy, and secondary outcomes.

Health behaviors	*n* (%)
Self‐reported dietary changes	
Low glycemic food selection	165 (43)
Portion control	105 (27)
Reduce sugar‐sweetened beverages	81 (21)
Reduced snacking	77 (20)
Reduced dining out/takeout	48 (13)
Advanced meal planning	39 (10)
Self‐reported physical activity changes	
Walking more	164 (43)
Going to the gym	86 (23)
Set step goals	30 (8)
Using the stairs	17 (4)

Obesity pharmacotherapy	
Medications prescribed on and off‐label, %	
Metformin	212 (56)
Topiramate	90 (24)
Bupropion	72 (19)
Phentermine	61 (16)
Liraglutide	44 (12)
Naltrexone HCL/bupropion HCL	9 (2)
Exenatide	7 (2)
Lorcaserin HCL^a^	7 (2)
Canagliflozin	4 (1)
Empagliflozin	3 (1)
Orlistat	2 (0.5)
Phentermine/topiramate	1 (0.3)
Zonisamide	1 (0.3)
Semaglutide^b^	0
Taking a combination of two or more individual medications	144 (38)

Secondary outcomes	
Self‐reported improvement at one or more visits	
Clothing size	103 (27)
Energy level	59 (15)
Sleep patterns	19 (5)
Joint pain	16 (4)
Clinical improvements at one or more visits	
Blood sugar	54 (14.1)
Blood pressure	53 (13.9)
Lipids	17 (5)

^a^ Withdrawn from the market in February 2020.

^b^ approved for treatment of Type II Diabetes in 2017 and obesity in June 2021.

### 3.2 Weight Trajectory Patterns

**FIGURE 1:**
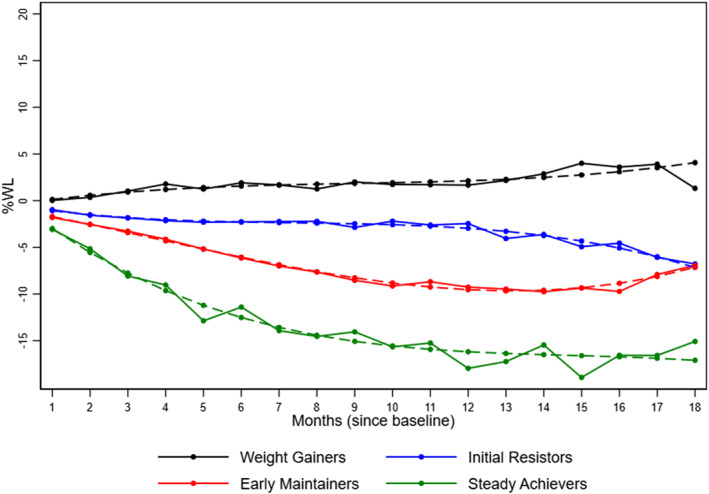
The mean percent of weight change from the initiation of the weight management program over 18 months was determined by the weight membership group. Group 1 (black line) termed Weight Gainers (*n* = 125); Group 2 (blue line) termed Initial Resistors (*n* = 150); Group 3 (red line) termed Early Maintainers (*n* = 70); and Group 4 (green line) termed Steady Achievers (*n* = 29).

Examination of weight trajectory patterns for percent weight loss resulted in a four‐group model presented in Figure 1. The groups were descriptively named as follows: (1) “Weight Gainers (WG)” included 33% (*n* = 125) of patients with an average weight gain of 1.3% of initial body weight (IBW) at 18 months; (2) “Initial Resistors (IR)” group had little to no weight loss for a prolonged period but meaningful weight loss between 12 and 18 months resulting in an average weight loss of 6.7% at 18 months; 40% (*n* = 150) of patients belonged to this group; (3) “Early Maintainers” (EM) reached their peak weight loss around 9 months and then remained at that weight through month 18 where their average weight loss was 7.1%; 19% (*n* = 70) of patients were in this group, and the (4) “Steady Achievers” (SA) group achieved 15% IBW. This group had the smallest percentage (7%; *n* = 29) of patients. Table 3 shows the characteristics of each group. Early Maintainers and Steady Achievers groups attended significantly more visits than the Weight Gainers and Initial Resistors (median number of visits: 5.5 and 5 vs. 2 and 1 visit, respectively) (*p* < 0.001) and had a higher proportion of individuals prescribed at least two AOMs (47% and 48% vs. 41% and 27% respectively; *p* = 0.014). In addition, “Steady Achievers” were significantly younger than “Early Maintainers” (median age: 43 vs. 55 years; *p* = 0.015).

**TABLE 3:** Participant characteristics of each weight change trajectory group at 18 months (*n* = 374).

	Weight Gainers (*n* = 125)	Initial resistors (*n* = 150)	Early maintainers (*n* = 70)	Steady achievers (*n* = 29)	*p*‐value
Age year, median (IQR)	50 (39, 59)	49 (38, 58)	54 (45, 63)	43 (34, 56)	0.015
Male, %	19	15	17	17	0.80
Non‐Hispanic Black race, %	46	42	34	24	0.12
Median baseline BMI kg/m^2^	39.6 (35.2, 45.4)	39.5 (35.8, 45.6)	38.6 (34.4, 42.3)	38.2 (34.9, 43.8)	0.59
Presence of metabolic syndrome, %	12	8	14	10	0.47
History of bariatric surgery, %	9	5	12	10	0.21
No. of weight management follow‐up visits, median (IQR)	1 (0,3)	2 (1,6)	5.5 (2,8)	5.0 (2,7)	< 0.001
Prescribed two or more AOMs simultaneously, %	27	41	47	48	0.014

**FIGURE 2:**
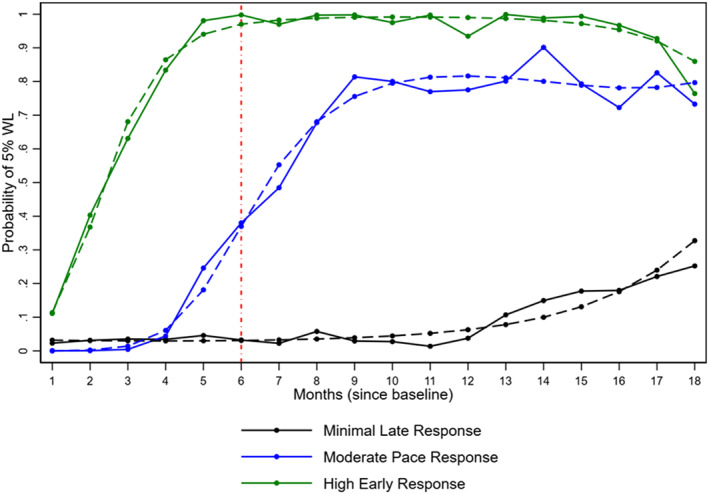
Probability of achieving at least 5% weight loss at 6 months (vertical red dash line) after initiating the weight management program. Group 1 (black line) termed Minimal late response (*n* = 247); Group 2 (blue line) termed Moderate Pace Response (*n* = 53); and Group 3 (green line) termed High Early Response (*n* = 74).

A three‐group model, presented in Figure 2, was the most suitable when examining the probability of achieving clinically meaningful weight loss. The groups were descriptively named as follows: (1) “Minimal Late Responders” included 66% (*n* = 247) patients; they had an extremely low probability in the first 6 months of achieving 5% weight loss; (2) “Moderately Paced Responders,” had an increasing probability of attaining 5% weight loss between months 4–6; 14% (*n* = 53) patients belonged to this group; and (3) the “High Early Responders” group 20% (*n* = 74) patients belonged to this group had a high probability of attaining the programmatic goal of 5% weight by 6 months. Patients in the three probability trajectories significantly differed by age (*p* = 0.015), non‐Hispanic Black race (*p* = 0.043), median number of follow‐up visits (*p* < 0.001), and proportion prescribed two or more anti‐obesity medications (*p* < 0.001) (Table 4).

**TABLE 4:** Participant characteristics for 5% weight loss trajectory groups (*n* = 374).

	Minimal late responders (*n* = 247)	Moderate pace responders (*n* = 53)	High early responders (*n* = 74)	*p*‐value
Age years, median (IQR)	50 (38, 58)	51 (41, 58)	52 (41, 62)	0.015
Male, %	16	19	18	0.88
Non‐Hispanic Black race, %	45	32	31	0.043
Median baseline BMI kg/m^2^	39.6 (35.4, 45.5)	39.5 (35.7, 46.4)	38.2 (34.7, 43.8)	0.59
Presence of metabolic syndrome, %	11	6	15	0.47
History of bariatric surgery, %	7	10	10	0.21
No. of follow‐up visits, median (IQR)	2 (0,5)	6 (2,9)	5 (2,7)	< 0.001
Prescribed two or more weight loss medications, %	33	60	41	< 0.001

The most initiated medication for the treatment of obesity was metformin 56% (*n* = 212), followed by topiramate 24% (*n* = 90), bupropion 19% (*n* = 70), and phentermine 16% (*n* = 61). Utilization of GLP1s (i.e., liraglutide and exenatide) accounted for 14% (*n* = 51) of the prescriptions.

## 4 Discussion

In a cohort of 393 patients in an integrated weight management primary care practice model, we identified four distinct weight loss trajectory groups over 18 months. The median percent weight loss (6.7%) of the Initial Resistor group (the largest of the four groups) was comparable to that of the Early Maintainers group (7.1%), albeit their trajectories were not the same, with the latter group experiencing weight regain after a year. The Weight Gainers, which represented the second largest of the groups (33%), experienced a 1.3% increase in weight by 18 months compared to the smallest group (7%), termed the Steady Achievers, who by month two, had already achieved 5% WL and lost the most weight (15%) of all groups by 18 months. Our probability model curves demonstrated that early weight loss (in the first months) was the most significant predictor of short (6 months) and long‐term weight loss (18 months). More importantly, membership in this group and the Early Maintainer's group was associated with attending more visits and being prescribed at least two medications at the same time. For this study, a combination pill (e.g., Qsymia) versus being prescribed the two active ingredients separately (e.g., phentermine and topiramate) was counted the same. Furthermore, younger age and non‐Hispanic Black race were associated with a lower probability of achieving clinically meaningful weight loss.

Our unique study focuses on identifying long‐term weight trajectory patterns in a diverse patient population receiving care in a real‐world weight management practice integrated into primary care. To the best of our knowledge, this is the first study to describe such patterns in a clinical population where more than half of the patients self‐reported race as non‐Hispanic Black (41%) or ethnicity as Hispanic (31%). Despite non‐Hispanic Black and Hispanic individuals being the groups with the highest rates of obesity in the United States, they are also the same groups with the lowest access to obesity medicine specialists [25]. Similar studies examining longitudinal weight change patterns in real‐world settings have reported cohorts where most participants self‐identified as White, underscoring the need for more research with diverse populations to improve the applicability of findings [13, 26, 27, 28, 29]. Furthermore, most research studies evaluating weight loss trajectories have been on web‐based behavioral interventions, with few studies examining the effects of behavioral interventions and anti‐obesity medications (AOMs) together on weight loss trajectories [13].

Our findings are consistent with previous research identifying two to seven distinct weight change patterns, from modest to significant weight loss. A study by Zhou et al. of a commercially based behavioral weight loss program identified four distinct weight loss patterns within the first 14 days that predicted long‐term weight loss success over 12 months. However, its methodology has limitations that may have introduced measurement and selection bias [26]. In a 12‐month web‐based coaching program in Finland, the authors identified five weight loss trajectories ranging from super‐responders who represented the smallest participant group (8%), losing an average of 15.7% of their IBW to the gainers (14.5% of participants) who slowly gained weight rather than losing it. In this latter group, participants gained about 3.5% of their IBW over 12 months [29]. Furthermore, the authors found that a higher baseline BMI (*p* < 0.001), more frequent weight entries (*p* = 0.004), and number of medications used for other chronic health conditions (*p* = 0.048) were associated with more significant mean weight loss. Overall, similar to our findings, these studies identified distinct weight trajectory patterns whereby early weight loss significantly predicted long‐term weight loss.

Group membership in our study was significantly associated with the number of visits attended and receipt of at least two AOMs prescribed simultaneously. The number of treatment sessions attended has been previously shown to lead to better outcomes in treating obesity [30]. The United States Preventive Services Task Force recommends that clinicians offer or refer patients with obesity to intensive (at least 12 sessions over 12 months) multicomponent behavioral interventions as a grade B recommendation [31]. Notably, this recommendation is devoid of the concurrent utilization of AOM. Our findings of a median number of five visits and AOM prescriptions resulting in clinically meaningful weight loss at 18 months suggest the need for more real‐world studies of combined treatment in high‐risk populations. While all FDA‐approved medications have been associated with significantly more weight loss compared to intensive behavioral changes alone [32, 33] they remain significantly underutilized, with only 2% of eligible adults with obesity being prescribed an AOM [34]. This underutilization is multifactorial, including a lack of physician training and insurance coverage for most AOMs.

The lack of coverage for AOMs by the Center for Medicaid and Medicare has a disproportionate impact on patients at the highest risk for the development and worsening of weight‐related health conditions. This is highly relevant to patients like our cohort, for whom Medicaid and Medicare insured more than half of the cohort. AOMs prescribing patterns for the cohort reflect a balance between dominant weight‐related health conditions and coverage barriers. The most common weight‐related health condition in the cohort was hypertension (58%). Based on the Framingham study, excess body weight accounted for 26% of cases of hypertension in men and 28% in women. Among all AOMs, phentermine is the most prescribed medication for weight loss worldwide and is an excellent low‐cost option for patients who do not have insurance coverage for AOMs. However, as the FDA label for phentermine hydrochloride (Adipex, Adipex‐P, Lomaira) warns against initiating therapy in patients with even mild hypertension, it subsequently becomes a medication avoided in a significant sample of patients. Similarly, Naltrexone‐Bupropion has been shown to increase blood pressure and is not recommended in patients with hypertension. With insurance coverage for all FDA‐approved medications, providers would have more safe and effective treatment options for patients with disease‐related contraindications.

Hence, as our results demonstrated, more than half of the patients in our practice were prescribed Metformin in an off‐label use format, with another third prescribed an FDA‐approved combination pill or separate prescriptions to achieve similar results to the combination pills. While the use of off‐label medications is a common practice, studies suggest that this trend is primarily due to the limited coverage of FDA‐approved drugs for specific patient groups [35, 36].

The presented findings have several limitations. The study used retrospective data abstracted from an electronic health record from a single academic primary care practice and may not be generalizable to other primary care settings. Data on adherence to dietary recommendations, physical activity, and AOMs were not objectively measured and were described solely based on patient self‐reports. Also, differentiating the weight trajectory patterns by medication classifications (e.g., metformin vs. liraglutide), medication dosage strength, or prescribing patterns (e.g., increase, decrease, change, discontinue) was not plausible due to missing data, which would have lowered the analytic sample size. Lastly, our analysis did not control for the presence of being on a GLP‐1 for the primary treatment of type 2 diabetes and only covers the period from 2012 to 2020 before the release of newer effective incretin‐based therapies, such as Semaglutide and Tirzepatide for the indication of weight loss [33, 37].

However, we hypothesize that if these medications were included in a future analysis, we would observe similar overall trends but potentially even more significant weight loss in a subgroup due to the increased potency and efficacy of the GLP‐1s. It was also not feasible to assess potential confounding variables that could impact the response to behavioral counseling or AOMS, including factors such as self‐efficacy, social support, and intrinsic motivation. Lastly, we acknowledge that race is a social construct and that our finding of an association between non‐Hispanic Black race and a lower probability of clinically meaningful weight loss reflects, in part, differences in social and economic advantages related to race or ethnicity. Nevertheless, our findings address a significant gap in obesity treatment research in a population at high risk of weight‐related health conditions.

Several strengths of this study are notable: a moderate‐sized sample comprised of a diverse population, a well‐described programmatic approach to a real‐world clinical practice setting, and an analysis that spanned 18 months, allowing observation of short‐term weight loss and weight loss maintenance patterns. Our findings support the need for more extensive studies to assess the effective adjunctive strategies and combination of medications to rescue those with limited to no weight loss by 2 months.

## Conflicts of Interest

The authors declare no conflicts of interest.
